# Differences in Aqueous Concentrations of Cytokines in Macular Edema Secondary to Branch and Central Retinal Vein Occlusion

**DOI:** 10.1371/journal.pone.0068149

**Published:** 2013-07-05

**Authors:** Jing Feng, Tong Zhao, Yan Zhang, Yan Ma, Yanrong Jiang

**Affiliations:** Department of Ophthalmology, People’s Hospital, Peking University, and Key Laboratory of Vision Loss and Restoration, Ministry of Education, Beijing, China; Massachusetts Eye & Ear Infirmary, Harvard Medical School, United States of America

## Abstract

**Purpose:**

This study investigates the differential aqueous concentrations of interleukin 6, 8, 1β (IL-6, IL-8, IL-1β, respectively), serum amyloid A (SAA), transforming growth factor (TGF)-β, basic fibroblast growth factor (bFGF), and vascular endothelial growth factor (VEGF) in eyes with macular edema as a result of a branch retinal vein occlusion (BRVO) or central retinal vein occlusion (CRVO).

**Principal Findings:**

Significantly higher concentrations of IL-6, IL-8, IL-1β, TGF-β, bFGF, SAA, and VEGF were found in the aqueous humor of CRVO and BRVO patients than in the aqueous humor of control patients. A significant correlation was observed between the concentration of bFGF and the inner central macular thickness (CMT) of BRVO patients (r = 0.688; P = 0.02). A significant correlation was observed between the concentration of SAA and both the full and outer CMT of the ischemic group (r = 0.545 and 0.683, respectively; P = 0.04 and 0.01, respectively). In the non-ischemic group, the level of IL-6 was significantly associated with inner CMT (r = 0.560; P = 0.03). The full and outer CMT was significantly reduced in CRVO patients when compared with BRVO patients (P = 0.02 and 0.02, respectively) after injection of intravitreal bevacizumab (IVB) at 4 weeks.

**Significance:**

Serum amyloid A as a major protein involved in the acute and chronic stages of inflammation, and IL-6 and bFGF were significantly associated with the extent of macular edema in patients with RVO. Besides VEGF, other inflammatory cytokines and angiogenesic factors may be associated with RVO. This finding may have implications for the medical treatment of RVO.

## Introduction

Retinal vein occlusion (RVO) is a prevalent retinal vascular disease, second only to diabetic retinopathy [1], [2]. RVOs primarily include central retinal vein occlusions (CRVOs) and branch retinal vein occlusions (BRVOs). In recent studies, the prevalence of RVO is estimated to be 5.2 per 1,000 patients [3]. Although CRVO accounts for only approximately 20% of RVOs, it leads to poorer visual acuity prognoses and quality of life when compared to patients with BRVO [4], [5]. There are several risk factors for CRVO, including patients over 65 years old, hypertension, smoking, atherosclerosis, and diabetes [6]. Macular edema, a severe, vision-threatening complication of CRVO, contributes greatly to a decreased quality of life [7]. Although panretinal laser coagulation is recommended for the treatment of neovascularization, the Central Vein Occlusion Study Group has not offered a unified understanding of macular edema [8].

Previous studies have demonstrated that angiogenic cytokines, such as vascular endothelial growth factor (VEGF), and many inflammatory cytokines, such as interleukin 6 (IL-6), IL-8, IL-12, IL-15, IL-17, and IL-23, are elevated in the ocular fluid of eyes affected by BRVO or CRVO when compared with control eyes [9]–[11]. The elevated expression of angiogenic cytokines (such as VEGF) and many inflammatory cytokines (including IL-6) has also been reported in the ocular fluid of patients with CRVO [12], [13]. However, little is known about the precise roles of these molecules in the pathogenesis of macular edema secondary to BRVO and CRVO. From a pathogenic perspective, decreased tissue perfusion and increased hydrostatic pressure within the involved segments may, as a consequence of the vascular obstruction, lead to intraretinal hemorrhages, exudation of fluid, varying levels of tissue ischemia, and eventual intraocular neovascularization if the retinal ischemia is pronounced [14].

Several therapeutic methods are used to treat macular edema. Macular grid laser photocoagulation is considered effective for the remission of macular edema; however, this treatment has provided only limited improvement of visual function [15]. Intravitreal anti-inflammatory therapy (triamcinolone acetonide, IVTA), intravitreal anti-VEGF (intravitreal bevacizumab or ranibizumab) therapy, and a combined therapy have been shown to be relatively safe and effective treatments for macular edema as a result of BRVO or CRVO [16]–[19]. However, inconsistent results have been obtained in recent comparative studies of intravitreal injections, and no exact guidelines exist for intravitreal injections.

Therefore, in this study, we compared the levels of angiogenic and inflammatory cytokines in the aqueous humor of eyes with macular edema secondary to BRVO or CRVO, and we evaluated the potential implications of these cytokines in the pathogenesis of BRVO and CRVO.

## Patients and Methods

The study was conducted in accordance with the Declaration of Helsinki, and we received approval from the Investigational Review Board of the People’s Hospital affiliated with Peking University. Informed consent for all examinations and procedures was obtained from the subjects. All participants provided their written informed consent to participate in this study.

### Study Subjects

Undiluted aqueous humor samples were collected from 10 eyes of 10 non-retinal disease patients (control group) with cataracts and 29 eyes of 29 RVO patients (study group) with macular edema, the latter including CRVO patients (18 eyes) and BRVO patients (11 eyes). The inclusion criteria for macular edema secondary to RVO were as follows: (1) decrease in visual acuity; (2) diffused macular edema as seen in fundus fluorescein angiography (FFA); and (3) a central macular thickness (CMT) of more than 250 µm, as measured by optical coherence tomography (OCT).The exclusion criteria were as follows: (1) treatment with intravitreal injection of corticosteroids or bevacizumab or panretinal photocoagulation within 6 months prior to the study; (2) vitreous hemorrhaging; (3) tractional retinal detachment; (4) previous ocular surgery; (5) glaucoma or ocular hypertension; and (6) macular edema caused by retinal conditions other than RVO.

All patients accepted intravitreal injection of 1.25 mg bevacizumab per 0.05 mL saline solution (Avastin; Genentech Inc., San Francisco, CA, USA). Before and after the treatment, all patients underwent ophthalmic examination, including best corrected visual acuity recording using manifest refraction and the logMAR visual acuity chart, non-contact tonometry, slit lamp biomicroscopy, ophthalmoscopy, and FFA, which was performed with a fundus camera (TRC-50EX; Tokyo Optical Co., Ltd., Tokyo, Japan). CMT was defined as the value of a 1 mm central area by OCT (Zeiss-Humphrey, Dublin, CA, USA) with the use of a macular thickness map from 6 radial scans that intersect at the fovea in the OCT retinal mapping program.

### Sample Collection

All injections and cataract surgeries were performed by the same surgeon (YR.J.) at the Peking University People’s Hospital. Aqueous humor was collected during intravitreal injection or cataract surgery. All procedures conformed to the Declaration of Helsinki for research involving human subjects. Informed consent was obtained from all participants. Undiluted aqueous humor samples (100 µL to 200 µL) were obtained through anterior chamber paracentesis. All injections and sample collections were performed by four physicians (J.F., T.Z., Y.Z., and Y.M.) with the use of a standard sterilization procedure that included topical povidone-iodine and levofloxacin drops. The aqueous samples were immediately frozen and stored in a sterilized plastic corning (2 mL; Corning Inc., Troy, MI, USA) at −80°C until use. The samples were assayed within 6 months of collection.

### Measurement Of Cytokines Via Multiplex Analysis

Aqueous concentrations of IL-6, IL-8, IL-1β, serum amyloid A (SAA), transforming growth factor (TGF)-β, basic fibroblast growth factor (bFGF), and VEGF were analyzed with the use of the Procarta Cytokine Assay Kit (Panomics Inc., Fremont, CA, USA). The assays used xMAP technology with multi-analyte profiling beads to detect and quantify multiple protein targets simultaneously, the detailed process of which was reported in a similar study [20].

### Statistical Analysis

A commercially available statistical software package (SPSS for Windows, version 17.0, SPSS Inc., Chicago, IL, USA) was used to perform statistical analysis of the data. The aqueous levels of IL-6, IL-8, SAA, IL-1β, TGF-β, bFGF, and VEGF are presented as mean ± standard deviation. A 1-sample Kolmogorov-Smirnov test was performed to examine whether the samples were distributed normally. Differences between the study group and the control group were estimated with a nonparametric Mann-Whitney rank sum test and *t* test when appropriate. Parameters were compared using Kruskal-Wallis H test to compare variables among various groups. Chi-square test and Fisher’s exact t test were used to compare noncontinuous variables. A Wilcoxon signed rank test was used for preinjection and postinjection clinical data. Pearson correlation test was used to explore the relationship between aqueous level of a cytokine and OCT parameters. Two-tailed probabilities of less than 0.05 were considered to indicate statistical significance.

## Results

The study included 36 patients, with 26 patients in the study group and 10 patients in the control group. Duration of symptoms, hypertension proportion, baseline best visual acuity, intraocular pressure, and baseline full and outer CMT did not vary significantly between the study group and the control group (Tables 1 and 2). Pearson correlation tests between age and concentrations of IL-6, IL-8, SAA, IL-1β, TGF-β, bFGF, and VEGF were performed to exclude age as a confounding factor. All correlation tests showed that age was not significantly associated with cytokine levels (P = 0.69, 0.13, 0.16, 0.43, 0.65, 0.38, and 0.31, respectively).

**Table 1:** Comparison of eyes with CRVO and BRVO.

Variables	CRVO (n = 18)	BRVO (n = 11)	Control (n = 10)	p value
**Age,years**	55.28±18.56	57.73±11.35	74.00±12.17	0.02^1^
**Gender(female%)**	13(72.2%)	5(45.5%)	1(10.0%)	0.01^2^
**Duration of symptoms, months**	8.53(1–24)	7.58(0.3–48)		0.07^3^
**With hypertension(%)**	8(44.4%)	6(54.5%)	4(40.0%)	0.84^2^
**Baseline visual acuity(logMAR)**	1.18±0.47	0.65±0.42	1.09±0.45	0.01^1^
**IOP(mmHg)**	15.68±4.58	12.52±3.61	13.67±0.47	0.15^1^
**Baseline full central macular thickness(µm)**	486.28±225.57	403.27±93.10		0.38^3^
**Baseline outer central macular thickness(µm)**	362.18±180.19	300.82±85.41		0.45^3^

Data are presented as the mean±standard deviation, number(%), or median(range). logMAR = Logarithm of the minimum angle of resolution.

^1^Kruskal-Wallis H test.

^2^Fisher’s exact test.

^3^Mann-Whitney U test.

CRVO = central retinal vein occlusion; BRVO = branch retinal vein occlusion.

**Table 2:** Comparison of eyes with non-ischemic and ischemic RVO.

Variables	Non-ischemic RVO (n = 15)	Ischemic RVO (n = 14)	Control (n = 10)	p value
**Age,years**	56.33±19.51	56.07±11.80	74.00±12.17	0.02^1^
**Gender(female%)**	9(60%)	9(64.29%)	1(10.0%)	0.02^2^
**Duration of symptoms, months**	6.57(1–24)	9.89(1.5–24)		0.74^3^
**With hypertension(%)**	7(46.7%)	7(50%)	4(40.0%)	0.92^2^
**Baseline visual acuity(logMAR)**	0.97±0.41	0.99±0.62	1.09±0.45	0.91^1^
**IOP(mmHg)**	14.73±5.36	14.21±3.34	13.67±0.47	0.98^1^
**Baseline full central macular thickness(µm)**	457.13±189.71	452.29±192.40		0.78^3^
**Baseline outer central macular thickness(µm)**	350.80±152.18	323.38±153.11		0.53^3^

Data are presented as the mean±standard deviation, number(%), or median(range). logMAR = Logarithm of the minimum angle of resolution.

^1^Kruskal-Wallis H test.

^2^Fisher’s exact test.

^3^Mann-Whitney U test.

RVO = retinal vein occlusion.

Our analyses of the aqueous cytokine levels showed significantly higher concentrations of IL-6, IL-8, IL-1β, TGF-β, bFGF, SAA, and VEGF in the eyes of patients with CRVO or BRVO than in the control eyes (Table 3). Higher concentrations of IL-6, IL-8, IL-1β, TGF-β, bFGF, SAA, and VEGF were also measured in the aqueous humor of patients with ischemic and non-ischemic RVO than in the control group (Table 4).

**Table 3:** Aqueous humor levels of cytokines (Log concentration pg/ml) in eyes with CRVO and BRVO.

Cytokines	CRVO group (n = 18)	BRVO group (n = 8)	p value	Total RVO (n = 28)	Control (n = 10)	p value
**IL-6**	0.96±0.24	0.96±0.20	0.91	0.96±0.23	0.41±0.08	<0.001*
**IL-8**	0.99±0.09	1.04±0.09	0.17	1.01±0.10	0.79±0.10	<0.001*
**SAA**	1.05±0.09	1.08±0.08	0.46	1.06±0.09	0.50±0.11	<0.001*
**IL-1β**	1.06±0.12	1.04±0.11	0.75	1.05±0.11	0.77±0.14	<0.001*
**TGF-β**	1.25±0.15	1.21±0.10	0.51	1.23±0.59	0.59±0.08	<0.001*
**bFGF**	1.38±0.26	1.21±0.57	0.40	1.32±0.42	0.73±0.11	<0.001*
**VEGF**	1.30±0.13	1.31±0.12	0.67	1.30±0.13	0.80±0.18	<0.001*

CRVO = central retinal vein occlusion; BRVO = branch retinal vein occlusion; IL = interlukin; SAA = serum amyloid A; TGF = transforming growth factor; bFGF = basic fiberblast growth factor; VEGF = vascular endothelial growth factor.

Independent-Samples T test.

* indicates P<.05.

**Table 4:** Aqueous humor levels of cytokines (Log concentration pg/ml) in eyes with ischemic and non-ischemic RVO.

Cytokines	Non-ischemic group (n = 18)	Ischemic group (n = 8)	p value	Total RVO (n = 28)	Control (n = 10)	p value
**IL-6**	0.97±0.23	0.95±0.24	0.80	0.96±0.23	0.41±0.08	<0.001*
**IL-8**	1.00±0.10	1.03±0.09	0.34	1.01±0.10	0.79±0.10	<0.001*
**SAA**	1.06±0.09	1.06±0.09	0.90	1.06±0.09	0.50±0.11	<0.001*
**IL-1β**	1.06±0.12	1.03±0.11	0.56	1.05±0.11	0.77±0.14	<0.001*
**TGF-β**	1.25±0.10	1.22±0.16	0.54	1.23±0.59	0.59±0.08	<0.001*
**bFGF**	1.30±0.34	1.33±0.51	0.83	1.32±0.42	0.73±0.11	<0.001*
**VEGF**	1.30±0.16	1.31±0.09	0.79	1.30±0.13	0.80±0.18	<0.001*

RVO = retinal vein occlusion; IL = interlukin; SAA = serum amyloid A; TGF = transforming growth factor; bFGF = basic fiberblast growth factor; VEGF = vascular endothelial growth factor.

Independent-Samples T test.

* indicates P<.05.

**Figure 1:**
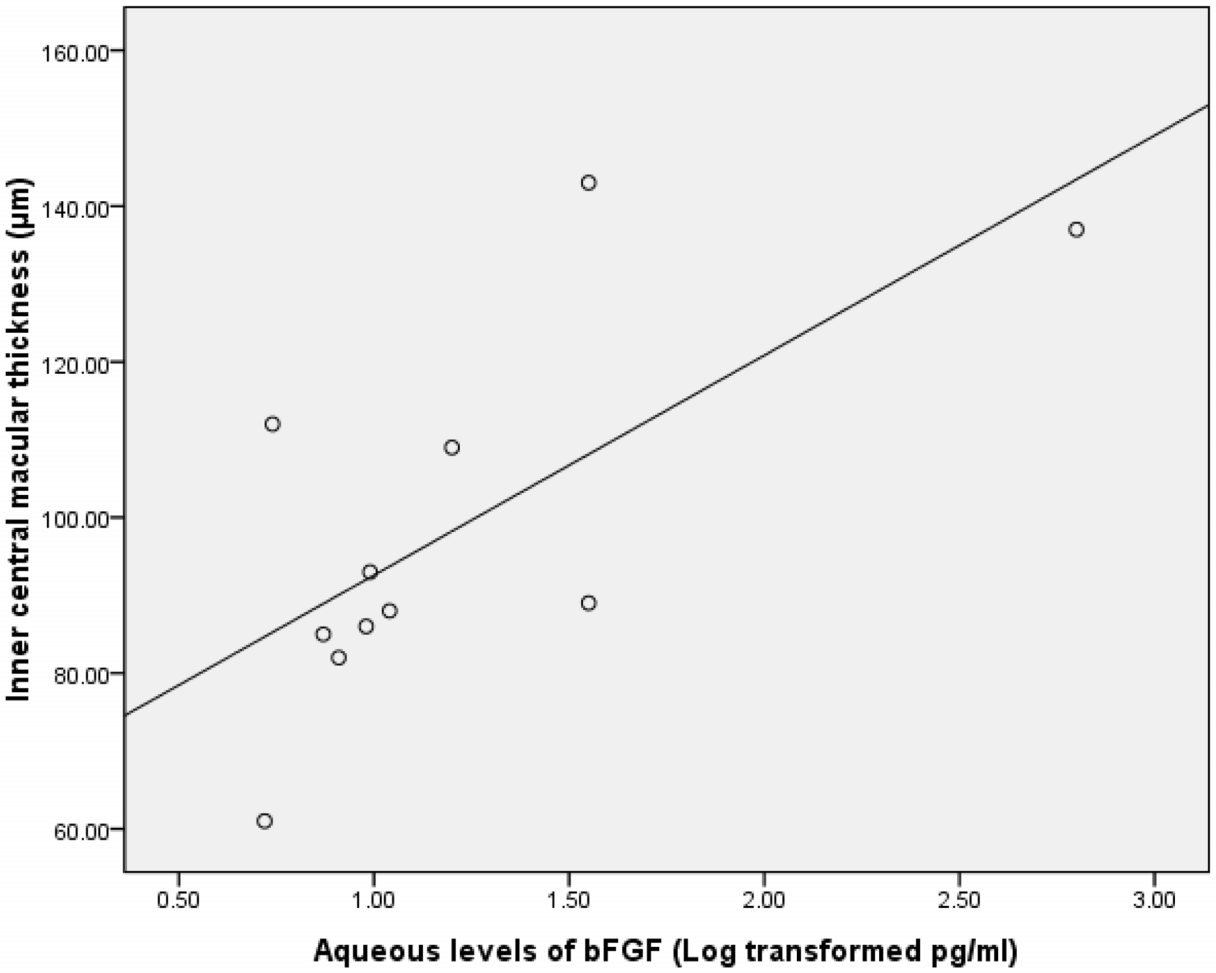
Scatterplot showing the association between the inner central macular thickness and the aqueous bFGF after the decadic logarithm transformation in in patients with macular edema resulting from BRVO, with a statistically significant correlation between the parameters(r = .688; P = .02).

**Figure 2:**
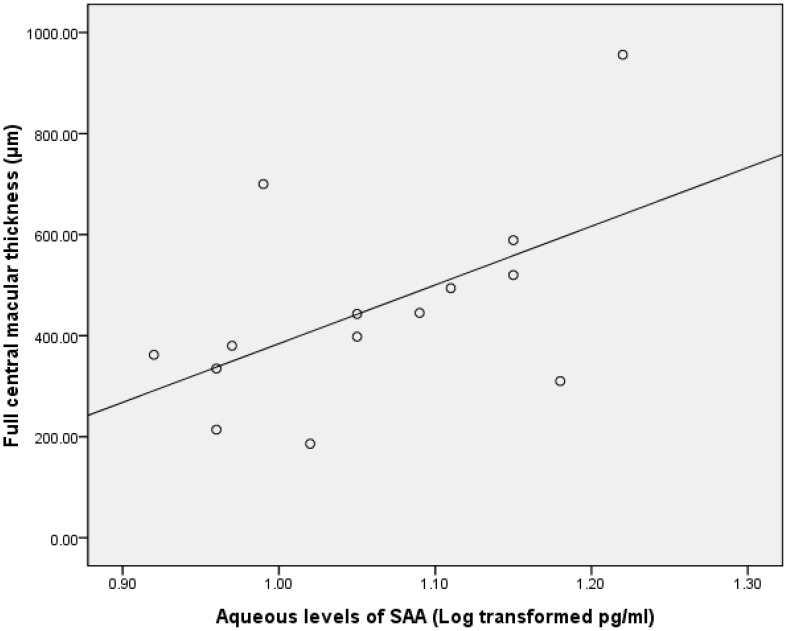
Scatterplot showing the association between the full central macular thickness and the aqueous SAA after the decadic logarithm transformation in in patients with macular edema resulting from ischemic RVO, with a statistically significant correlation between the parameters(r = .545; P = .04).

**Figure 3:**
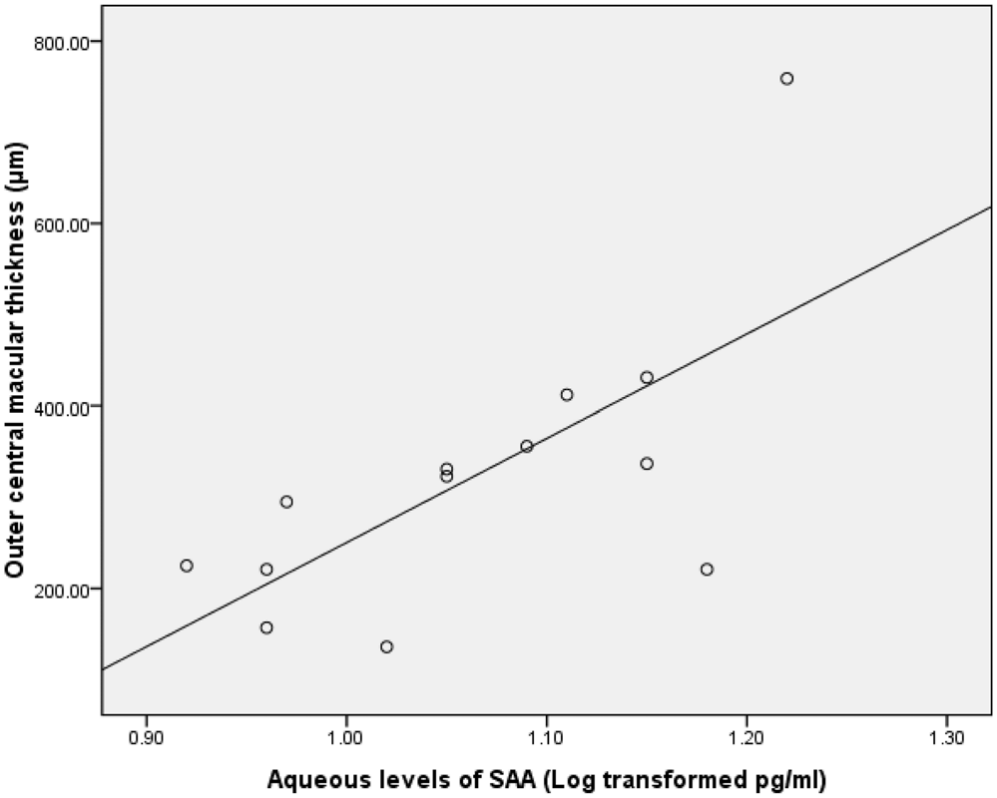
Scatterplot showing the association between the outer cental macular thickness and the aqueous SAA after the decadic logarithm transformation in in patients with macular edema resulting from ischemic RVO, with a statistically significant correlation between the parameters(r = .683; P = .01).

**Figure 4:**
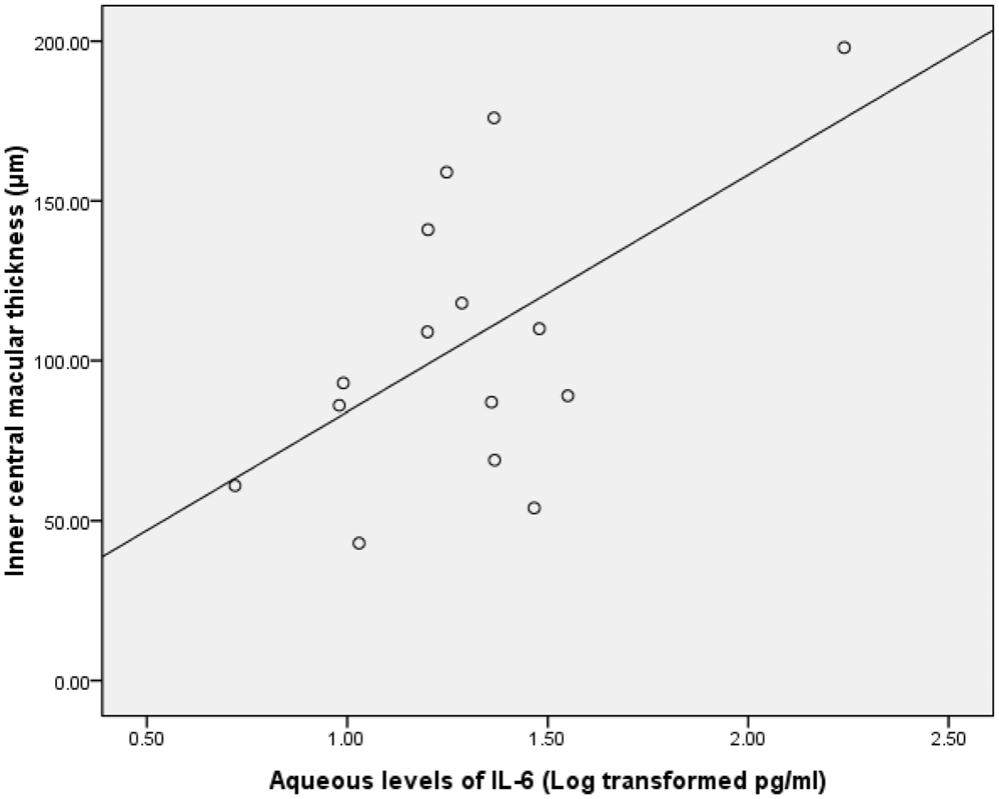
Scatterplot showing the association between the inner cental macular thickness and the aqueous IL-6 after the decadic logarithm transformation in in patients with macular edema resulting from non-ischemic RVO, with a statistically significant correlation between the parameters(r = .560; P = .03).

Data on the aqueous concentration of cytokines did not show a normal distribution according to a Gaussian distribution curve. Therefore, we performed Pearson correlation test on the transformed data of a decadic logarithm scale. The intraocular concentration of bFGF was significantly correlated with inner CMT in the group with BRVO (r = 0.688; P = 0.02; Table 5, Figure 1). Furthermore, the level of SAA in the ischemic group was significantly associated with both full and outer CMT (r = 0.545 and 0.683, respectively; P = 0.04 and 0.01, respectively; Table 6, Figure 2 & 3). The intraocular concentration of IL-6 was significantly correlated with the inner CMT in the non-ischemic group (r = 0.560; P = 0.03; Table 6, Figure 4).

**Table 5:** **P** values of each Pearson correlation test between level of cytokines and OCT parameters in CRVO and BRVO groups.

	CRVO			BRVO		
Cytokines	Full CMT	Inner CMT	Outer CMT	Full CMT	Inner CMT	Outer CMT
**IL-6**	.42	.33	.64	.17	.08	.48
**IL-8**	.77	.38	.63	.73	.54	.73
**SAA**	.13	.14	.08	.76	.80	.47
**IL-1β**	.50	.44	.53	.35	.15	.14
**TGF-β**	.70	.62	.83	.75	.11	.54
**bFGF**	.30	.19	.47	.40	.02* r = .688	.78
**VEGF**	.64	.90	.38	.59	.62	.77

CMT = central macular thickness; CRVO = central retinal vein occlusion; BRVO = branch retinal vein occlusion.

* indicates P<.05.

**Table 6:** P values of each Pearson correlation test between level of cytokines and OCT parameters in ischemic and non-ischemic RVO.

	Ischemic			Non-ischemic		
Cytokines	Full CMT	Inner CMT	Outer CMT	Full CMT	Inner CMT	Outer CMT
**IL-6**	.94	.39	.72	.11	.03* r = .560	.17
**IL-8**	.55	.84	.83	.64	.50	.70
**SAA**	.04* r = .545	.10	.01* r = .683	.77	.90	.68
**IL-1β**	.26	.72	.26	.63	.72	.62
**TGF-β**	.78	.93	.44	.63	.29	.77
**bFGF**	.34	.32	.90	.48	.76	.33
**VEGF**	.45	.98	.75	.30	.80	.22

CMT = central macular thickness; RVO = retinal vein occlusion.

* indicates P<.05.

Full, outer, and inner CMT significantly decreased in the CRVO group, while only full and outer CMT decreased significantly in the BRVO group. However, no significant change was observed between the two groups (P = 0.45, 0.65, and 0.22, respectively; Table 7). Likewise, the full and outer CMT in the ischemic group significantly decreased at 4 weeks after injection when compared with the non-ischemic group (P = 0.02 and 0.012, respectively; Table 8).

**Table 7:** Changes in Central Macular Thickness of CRVO and BRVO.

	Full CMT			Outer CMT			Inner CMT		
	Pre-injection	Post-Injection	P Value	Pre-injection	Post-injection	P Value	Pre-injection	Post-injection	P Value
**CRVO**	486.28±225.57	306.46±103.45	0.003*	362.18±180.19	219.08±75.50	.002*	111.47±50.42	85.54±34.64	.008*
**BRVO**	403.27±93.10	300.60±79.20	.008*	300.82±85.41	219.80±61.39	.005*	98.64±23.36	80.9±18.68	.17

CMT = central macular thickness; CRVO = central retinal vein occlusion; BRVO = branch retinal vein occlusion.

Wilcoxon signed rank test.

* indicates P<.05. Compared between CRVO and BRVO groups. P = .45; P = .65; P = .22.

**Table 8:** Changes in Central Macular Thickness of ischemic and non-ischemic RVO.

	Full CMT			Outer CMT			Inner CMT		
	Pre-Injection	Post-injection	P Value	Pre-injection	Post-injection	P Value	Pre-injection	Post-injection	P Value
**Ischemic**	452.29±192.40	297.88±68.11	.01*	323.38±153.11	218.38±50.72	.01*	106.69±40.63	79.63±22.21	.05
**Non-ischemic**	457.13±189.71	307.13±104.72	.002*	350.80±152.18	219.93±77.98	.001*	106.20±43.86	85.60±31.70	.03

CMT = central macular thickness; RVO = retinal vein occlusion.

Wilcoxon signed rank test.

* indicates P<.05. Compared between ischemic and non-ischemic groups. P = .02; P = .02; P = .13.

## Discussion

Recent studies have investigated intraocular cytokine levels in RVO patients [9]–[13]. However, only one intraocular cytokine analysis compared diabtetic macular edema (DME) and RVO directly, and no discussion concerning macular edema resulting from BRVO and CRVO has occurred [21]. The current study is the first report to compare the aqueous samples of macular edema as a result of BRVO or CRVO, consequently comparing the intraocular cytokines of macular edema resulting from ischemic and non-ischemic RVOs.

This study showed that intraocular IL-6, IL-8, IL-1β, TGF-β, bFGF, SAA, and VEGF were strikingly higher in patients with macular edema than in the control patients, regardless of whether this was due to CRVO or BRVO. We also showed that the aqueous SAA level was statistically significantly associated with both full and outer CMT in ischemic RVO patients. The intraocular level of bFGF was significantly correlated with the inner CMT in the group with BRVO. Furthermore, the level of IL-6 in the non-ischemic group was significantly associated with inner CMT.

The results indicated that significantly higher concentrations of IL-6, IL-8, IL-1β, TGF-β, bFGF, SAA, and VEGF were found in the eyes of CRVO and BRVO patients than in the control eyes. Both inflammatory and angiogenic factors were therefore involved in the development of macular edema secondary to RVO. These results are consistent with those of previous studies. Although RVO is not a classic inflammatory disease (e.g. uveitis), evidence either from anatomic studies or molecular experiments supports the hypothesis that inflammation affects disease pathogenesis and progression [9]–[13].

SAA is a classic acute phase protein that responds to injury, infection, inflammation and neoplasia [22]. Although SAA is produced mainly by hepatocytes under the appropriate stimulation, extrahepatic SAA synthesis has also been implicated (via monocytes, endothelial cells, fibroblasts, etc.) in the pathogenesis of several chronic inflammatory diseases, including atherosclerosis, Alzheimer disease, inflammatory arthritis, and several cancer variants [23], [24]. Our previous study also found that vitreous SAA and IL-6 levels in the eyes of proliferative diabetic retinopathy (PDR) patients were significantly higher than in nondiabetic patients, a result indicating that an inflammatory process may be involved in the development of PDR [25]. In a recent study, we found that the aqueous SAA level was statistically significantly associated with both full and outer CMT in ischemic RVO patients. This finding suggests that SAA potentially plays a role in the development of ischemic RVO. Our previous study indicated that SAA may be produced by retinal vascular endothelial cells and fibrillar structures in the eye [25]. The present study further revealed that SAA may be involved in other retinal diseases, such as RVO. Nevertheless, the question of how SAA acts in inflammation or angiogenesis during the development of RVO needs to be explored.

IL-6 is a cytokine derived from activated T lymphocytes with multiple functions, including induction of B-cell growth, induction of B-cell differentiation and antibody production, induction of differentiation and proliferation of T cells, synergistic induction with IL-3 for hematopoietic cell growth, and induction of the hepatocyte secretion of acute-phase inflammatory proteins [26], [27]. IL-6 is also known to increase vascular permeability and angiogenesis by inducing the expression of VEGF [28]. The role of IL-6 in inflammation in DME and macular edema due to RVO has already been reported in several studies [9]–[13]. In a previous study, Won June Lee et al. reported that aqueous levels of IL-6 in the BRVO with macular edema (BRVO-ME) group were not significantly different when compared with controls and were significantly lower when compared with the levels in the diabetic macular edema group, which indicates that the role of inflammation in BRVO-ME is less influential than in DME [21]. In our study, the level of IL-6 in the non-ischemic RVO group was significantly associated with inner CMT. The results of several recently reported studies comparing the treatment effects of IVTA in macular edema resulting from BRVO were also consistent with our findings. Ding et al. reported that IVT showed better efficacy 2 weeks after injection than intravitreal bevacizumab (IVB) [19]. This could be explained by the possibility that many inflammatory cytokines (not just VEGF) play a major role in the pathogenesis of RVO.

Recently, many studies have proven the efficacy and safety of the intravitreal use of bevacizumab for macular edema due to RVO [16]–[19]. In our study, we also showed the remarkable effects on macular thickness improvement at 4 weeks after a single anti-VEGF therapy. Although strong evidence indicates a causative role of VEGF in retinal neovasculation (NV), other angiogenic factors most likely stimulate NV in a parallel and concerted fashion.The finding in our study that the intraocular level of bFGF was significantly correlated with inner CMT in the patients with macular edema due to BRVO has never been reported. Members of the fibroblast growth factor (FGF) family, such as bFGF, have been implicated for many years in the development of retinal NV [29]–[31]. Macular edema due to RVO is believed to be associated with increased venous and capillary pressure, breakdown of the blood–retinal barrier, and enhanced vessel permeability. Hypoxia-induced upregulated expression of VEGF plays an important role in this course [32], [33]. Zittermann et al. suggested that bFGF may be an important positive regulator of leukocyte recruitment in acute and chronic inflammation, which could increase vascular permeability [34]. Therefore, one may infer that the increased aqueous bFGF levels of BRVO patients may be involved in macular edema through increased vascular permeability.

The potential limitations of our study should also be mentioned. First, it is inaccurate to assume that a particular cytokine affects pathogenesis on the simple basis of measuring elevated aqueous levels. A particular cytokine is released as a result of the disease process. Thus, it cannot be the cause of a disease process. Second, we could not control all possible confounding variables, such as time from onset. These can affect cytokine levels in the eye. Third, our sample size was small. Thus, a large, multicenter, randomized, prospective study is required to clarify the pathogenesis of macular edema secondary to RVO as associated with cytokines. This study could serve as the basis for future research that involves a large number of eyes and thus could enable the development of disease-specific treatments.

In conclusion, besides VEGF, other inflammatory cytokines and angiogenesis factors may be associated with RVO. This finding may have implications for the medical treatment of RVO.
